# Effect of osteopathic manipulative treatment on gastrointestinal function and length of stay of preterm infants: an exploratory study

**DOI:** 10.1186/2045-709X-19-15

**Published:** 2011-06-28

**Authors:** Gianfranco Pizzolorusso, Patrizia Turi, Gina Barlafante, Francesco Cerritelli, Cinzia Renzetti, Vincenzo Cozzolino, Marianna D'Orazio, Paola Fusilli, Fabrizio Carinci, Carmine D'Incecco

**Affiliations:** 1 EBOM - European Institute for Evidence Based Osteopathic Medicine, viale Unità d'Italia 1, 66100 Chieti, Italy; 2 AIOT - Accademia Italiana Osteopatia Tradizionale, via Prati 29, 65124 Pescara, Italy; 3 Unità di Terapia Intensiva Neonatale - Ospedale Civile Spirito Santo, Via Renato Paolini 45, 65124 Pescara, Italy

## Abstract

**Background:**

Organizational improvement of neonatal intensive care units requires strict monitoring of preterm infants, including routine assessment of physiological functions of the gastrointestinal system and optimized procedures for the definition of appropriate discharge timing.

**Methods:**

We conducted a prospective study on the effect of osteopathic manipulative treatment in a cohort of N = 350 consecutive premature infants admitted to a neonatal intensive care unit without any major complication between 2005 and 2008. In addition to ordinary care, N = 162 subjects received osteopathic treatment. Endpoints of the study were differences between study and control groups in terms of excessive length of stay and gastrointestinal symptoms, defined as the upper quartiles in the distribution of the overall population. Statistical analysis was based on crude and adjusted odds ratios from multivariate logistic regression.

**Results:**

Baseline characteristics were evenly distributed across treated/control groups, except for the rate of infants unable to be oral fed at admission, significantly higher among those undergoing osteopathic care (p = .03). Osteopathic treatment was significantly associated with a reduced risk of an average daily occurrence of gut symptoms per subject above .44 (OR = 0.45; 0.26-0.74). Gestational age lower or equal to 32 weeks, birth weight lower or equal to 1700 grams and no milk consumption at admission were associated with higher rates of length of stay in the unit of at least 28 days, while osteopathic treatment significantly reduced such risk (OR = 0.22;0.09-0.51).

**Conclusions:**

In a population of premature infants, osteopathic manipulative treatment showed to reduce a high occurrence of gastrointestinal symptoms and an excessive length of stay in the NICU. Randomized control studies are needed to generalize these results to a broad population of high risk newborns.

## Background

Significant improvements in neonatal technology utilized in neonatal intensive care units (NICUs) over the last 2 decades, along with evidence-based care guidelines, have significantly improved hospitalization and survival for both low birth weight (LBW) infants and the residual preterm population, albeit at a high cost. A major proportion of pediatric hospital stays in the United States is attributable to neonatal conditions that rank among the most expensive items in the list of services provided for children [1]. The average cost per infant is highest for preterm newborns with gestational age (GA) between 24-31 weeks, and next highest for those between 32-36 weeks, as opposed to the general population [2]. Costs per surviving infant generally decrease with increasing GA. In the United States, preterm/LBW infants account for half the hospitalization costs of all newborns and one quarter of overall pediatric costs [3].

Length of stay (LOS) in NICUs is strongly associated with GA and birth weight [4]. Infants delivered at the earliest GA have the longest hospital stays, partly because of the higher incidence of medical complications in very low birth weight (VLBW) infants.

However, compared to term infants, premature infants are unique in their need to attain not only medical stability but also physiologic maturity, including adequate temperature control, cessation of apnea and bradycardia, and adequate feeding behavior, before they are safely discharged to home [5,6].

Patterns of hospitalization of preterm infants are also associated with the presence of clinical symptoms of abnormal gastrointestinal function. In particular, vomit and regurgitation were found to be associated with increased esophageal acid occurrence among NICU patients [7], as well as gastric residuals (GR) [8], which can be linked to feeding behaviors and definitely improved by targeted feeding strategies.

In VLBW infants, feeding tolerance algorithms are based on pre-prandial GR volume measurement. High pre-prandial volumes of GR are regarded as significant markers of feeding intolerance [9]. Previous studies in NICUs show that neonates under stress have a higher incidence of stress-induced gastric mucosal damage [10,11]. Functional constipation and hard stools are common conditions in both term and preterm infants, usually leading to changes in feeding formulas [12] and use of enemas in specific settings [13].

Non invasive treatments to improve feeding tolerance and to reduce clinical complications of premature infants may represent a convenient option in the absence of standard procedures for specific subgroups of patients.

The present report describes the activity of a research team investigating the effects of Osteopathic Manipulative Treatment (OMT) in preterm infants, including monitoring of physiological functions of the gastrointestinal system and LOS.

## Methods

### Objective and endpoints of the study

To evaluate the efficacy of OMT on premature infants during hospitalization. Endpoints of the study were differences between study and control groups in terms of changes in gastrointestinal function and LOS.

Primary endpoints were measured over the entire period of NICU hospitalization as follows:

I. High frequency of gut symptoms, defined as the upper quartile of the average number of episodes of vomit, regurgitation, GR and enema per measurement visit per subject.

II. Excess duration of LOS, defined as the upper quartile of LOS in NICU per subject.

### Study Design and Population

The study was based on a non randomized, longitudinal observational design investigating outcomes in a cohort of newborns admitted to the NICU of the main public hospital in Pescara, Abruzzo, Italy.

Eligible subjects included all infants consecutively admitted between January 2005 to June 2008 (N = 663). A total of N = 359 passed the following exclusion criteria: GA less than 29 weeks, or greater than 37 weeks; osteopathic treatment performed more than 14 days after birth; newborn transferred to/from other hospital/unit; newborn from an HIV seropositive and/or drug addicted mother; newborn with any of the following clinical conditions: genetic disorders, congenital abnormalities, cardiovascular abnormalities, neurological disorders; proven or suspected necrotizing enterocolitis with or without gastrointestinal perforation; proven or suspected abdominal obstruction; pre- and/or post- surgery patients; pneumoperitoneum and/or atelectasis. Among the 304 subjects excluded, 232 infants had a GA below 29 or above 37, while 78 subjects presented with severe clinical conditions.

After enrollment, 4 additional infants were dropped because of an unrecorded birth weight, and 5 infants (2 from the study group; 3 from the control group) because of complications arising during hospitalization.

The final total number of infants analyzed in this study was 350.

A total of 188 preterm infants were non-randomly assigned to routine neonatal care; while 162 subjects received routine care plus OMT. All patients from both groups were transferred from the delivery and/or operating room to the NICU immediately after birth.

No prior manipulation provided by any physical and/or massage therapist was performed on any infant.

### Data collection

Data collection was performed by undergraduate osteopaths from the Accademia Italiana Osteopatia Tradizionale (AIOT). Measurements were recorded twice a week (Tuesdays and Fridays) based upon NICU's clinical charts completed by nursing staff who provided care on the same day.

Additional infant information was included: date of birth, admission/discharge from NICU, GA at birth (based on best obstetrical estimate), birth weight at admission and discharge, formula and/or breast milk intake volume. Gastrointestinal function was measured as regurgitation (defined as the passage of refluxed gastric contents into the oral pharynx), or vomiting (defined as the expulsion of the refluxed gastric contents from the mouth, i.e. feeding tolerance), or GR finding (milky, bilious and bloody; measured only on infants with oro/naso-gastric tube, recorded as present/not present), frequency of stooling and enema administration per patient care encounter. A neurological/developmental evaluation at entry/discharge was not available for this study as it does not constitute part of routine assessment in the NICU.

Data were directly entered on an Excel spreadsheet

### Osteopathic Manipulative Treatment

Osteopathic treatment was administered to the intervention group on Tuesdays and Fridays. Subjects in the study arm received osteopathic care within 14 days after birth, regardless of the application of any other procedure (i.e. mechanical ventilation, blood transfusion or phototherapy).

OMT was performed by a group of osteopaths certified by the Registro degli Osteopati d'Italia with at least five years of clinical experience.

Treatment duration ranged between 20-30 minutes. The infant's entire body was evaluated and manipulative procedures were provided as indicated by the osteopathic palpatory structural examination results. Osteopaths performing OMT were trained to use only indirect and fluidic techniques which included: indirect myofascial, sutural spread, balanced membranous tension and balanced ligamentous tension (according to teachings of William Garner Sutherland, DO, and others [14]).

### Clinical procedures and discharge strategy

Feeding regimen, feeding strategies and enema administration were based on the application of standard international guidelines to both study arms [13,15]. As distinct from UK/US hospitals, enema prescription used by the study NICU included five percent glucose glycerin enemas (10:1 mixture, 5 mL/kg), administered twice a day, until infants spontaneously expel at least 1 stool per day.

Physiological conditions required for discharge included: maintenance of body heat at room temperature, coordinated sucking, swallowing, and breathing while feeding; sustained pattern of weight gain; and stability of cardiorespiratory function (no episodes of apnea/bradycardia for 2-5 days, free of supplemental oxygen support) [6].

### Statistical analysis

Main results are expressed in terms of odds ratios between each level of a potential risk factor and a set reference category (R.C.), with primary endpoints classified as binary outcomes (low/high).

Potential confounders included the following characteristics (categories): gender, GA (≤ 32; > 32- ≤ 35; > 35 weeks), birth weight (≤ 1700; > 1700- ≤ 2200; > 2200 grams), oral feeding at admission (No/Yes).

Univariate statistical tests included formal tests of the differences between study and control groups using chi-square for categorical variables and unpaired t-tests for continuous measurements.

Multivariate logistic regression was used to estimate the independent effect of OMT on primary outcomes, simultaneously adjusting for all potential confounders. Statistical significance was based on a probability level (α) equal to 0.05. Results were expressed in terms of point estimates (odds ratios: OR) and 95% confidence intervals (C.I.). All analyses were performed using the statistical programming language R [16].

## Results

Univariate statistical analyses are shown in Table 1. No significant imbalances were found among treated and control groups in terms of main characteristics measured at admission, except for milk at admission (p = 0.03), showing a higher percentage of infants unable to be oral fed at entry into this study among those treated with OMT.

**Table 1:** General characteristics of the study population

	Study Group	Control Group	p value
N	162 (46.3)	188 (53.7)	
Gender			
Females	81 (50.0)	89 (47.3)	
Males	81 (50.0)	99 (52.7)	0.70
Gestational Age			
≤ 32	39 (24.1)	43 (22.9)	
> 32, ≤ 35	69 (42.6)	72 (38.3)	
> 35	54 (33.3)	73 (38.8)	0.56
Weight (grams)			
At Birth			
≤ 1700	27 (16.7)	36 (19.2)	
> 1700, ≤ 2200	62 (38.3)	63 (33.5)	
> 2200	73 (45.0)	89 (47.3)	0.62
At Admission*	2148 (486.7)	2212 (562.3)	0.25
Oral feeding at admission			
No	129 (79.6)	129 (68.6)	
Yes	33 (20.4)	59 (31.4)	0.03

Numbers in Table are N (%), p values from Chi Square test

* = mean, (standard deviation); p value from t test

Upper quartiles led to the definition of the following thresholds for the outcomes of interest:

1) average daily occurrence of gut symptoms per subject above .44;

2) LOS of at least 28 days.

Results for gastrointestinal function are shown in Table 2. None of the risk factors considered as potential correlates were found to be associated with an high rate of gut symptoms, except for OMT (OR = 0.45;0.27-0.74). Multivariate logistic regression confirmed OMT to be independently associated with a 55% reduction of gastrointestinal symptoms (Adjusted OR = 0.45;0.26-0.74).

**Table 2:** Results for Average Daily Gut Symptoms: Crude and Adjusted Odds Ratios from Multivariate Logistic Regression

	Average Daily Gut Symptoms*		**Univariate O.R**.		Adjusted O.R	
	≤ 0.44	> 0.44	O.R. (95%CI)	p > |χ^2^	O.R. (95%CI)	p > |χ^2^
	
N	262 (74.9)	88 (25.1)				
Gender						
Females [R.C]	129 (75.9)	41 (24.1)	1	-	1	-
Males	133 (73.9)	47 (26.1)	1.11 (0.68-1.80)	0.759	1.08 (0.65-1.79)	0.777
Gestational Age						
≤ 32	57 (69.5)	25 (30.5)	1.20 (0.65-2.21)	0.670	1.02 (0.43-2.40)	0.965
> 32, ≤ 35	112 (79.4)	29 (20.6)	0.71 (0.40-1.25)	0.293	0.72 (0.39-1.32)	0.292
> 35 [R.C]	93 (73.2)	34 (26.8)	1	-	1	-
Birth Weight (grams)						
≤ 1700	39 (67.2)	19 (32.8)	1.54 (0.80-2.96)	0.265	1.39 (0.55-3.46)	0.481
> 1700, ≤ 2200	100 (76.9)	30 (23.1)	0.95 (0.55-1.63)	0.952	1.03 (0.55-1.93)	0.927
> 2200 [R.C]	123 (75.9)	39 (24.1)	1	-	1	-
Oral feeding at admission						
No	192 (74.4)	66 (25.6)	1.09 (0.63-1.90)	0.860	1.18 (0.67-2.13)	0.583
Yes [R.C]	70 (76.1)	22 (23.9)	1	-	1	-
OMT						
No [R.C]	128 (68.1)	60 (31.9)	1	-	1	-
Yes	134 (82.7)	28 (17.3)	0.45 (0.27-0.74)	0.002	0.45 (0.26-0.74)	0.002

R.C. = Reference Category

* No. of episodes of Vomit, Regurgitation, Gastric residual and Enema

Results for LOS are reported in Table 3. Univariate odds ratios showed the following categories to be associated with increased rates of LOS equal or above 28 days: GA ≤ 32 weeks (OR = 38.10;16.40-88.20; R.C.:GA > 35 weeks), birth weight ≤ 1700 gm vs > 2200 gm (OR = 120.60;42.70-340.60) and birth weight > 1700 gm, ≤ 2200 gm (OR = 5.80;2.40-13.80; R.C.: birth weight > 2200 gm), oral feeding at admission (OR = 2.85;1.44-5.66) and OMT (OR = 0.51;0.30-0.85). Multivariate logistic regression showed similar patterns, confirming an independent effect of OMT, simultaneously adjusted for all factors, corresponding to more than a 75% reduction in excessive LOS (Adjusted OR = 0.22;0.09-0.51)

**Table 3:** Results for Length of Stay (LOS): Crude Odds Ratios (p value from Cochran Mantel Haenszel Chi Square Test of Zero Correlation) and Adjusted Odds Ratios from Multivariate Logistic Regression (p value from partial test on regression coefficient)

	LOS (days)		**Univariate O.R**.		**Adjusted O.R**.	
	< 28	≥ 28	O.R. (95%CI)	p > |χ^2^	O.R. (95%CI)	p > |χ^2^
	
N	267 (76.3)	83 (23.7)				
Gender						
Females [R.C]	128 (75.3)	42 (24.7)	1	-	1	-
Males	139 (77.2)	41 (22.8)	0.90 (0.55-1.47)	0.765	1.40 (0.63-3.10)	0.412
Gestational Age						
≤ 32	21 (25.6)	61 (74.4)	38.10 (16.40-88.20)	< 0.001	10.90 (3.53-33.72)	< 0.001
> 32, ≤ 35	128 (90.8)	13 (9.2)	1.33 (0.55-3.22)	0.680	0.76 (0.27-2.15)	0.609
> 35 [R.C]	118 (92.9)	9 (7.1)	1	-		
Birth Weight (grams)						
≤ 1700	9 (15.5)	49 (84.5)	120.60 (42.70-340.60)	< 0.001	43.23 (11.63-160.66)	< 0.001
> 1700, ≤ 2200	103 (79.2)	27 (20.8)	5.80 (2.40-13.80)	< 0.001	3.01 (1.05-8.68)	0.041
> 2200 [R.C]	155 (95.7)	7 (4.3)	1	-	1	-
Oral feeding at admission						
No	186 (72.1)	72 (27.9)	2.85 (1.44-5.66)	0.003	3.11 (1.05-9.25)	0.041
Yes [R.C]	81 (88.0)	11 (12.0)	1	-	1	-
OMT						
No [R.C]	133 (70.7)	55 (29.3)	1	-	1	-
Yes	134 (82.7)	28 (17.3)	0.51 (0.30-0.85)	0.012	0.22 (0.09-0.51)	< 0.001

R.C. = Reference Category

## Discussion

The main objective of this exploratory study was to investigate the effects of OMT in a population of premature infants in terms of gastrointestinal functions and LOS.

The medical literature lacks information of any potential benefits of complementary treatments in this area. To the best of the authors' knowledge, OMT in premature newborns has never been documented by pediatric specialty journals. Studies carried out in pediatric patients suggested positive effects of OMT in very young children [17-19]. In the broader field of manual therapy, specialists of massage therapy and kinesthetic stimulation showed positive results in premature infants [20]. However, such findings were inconsistent and obtained with heterogeneous methods, showing only minimal differences in terms of clinical significance [20,21].

The present study suggests that OMT may reduce the occurrence of frequent symptoms of abnormal gastrointestinal functionality.

Precise mechanisms for such positive effects generated by OMT are difficult to specify, but several hypotheses can be offered on the basis of neurological, tissue and neuroendocrine factors.

In terms of neurology, there is evidence of an association between autonomic nervous system function and OMT, showing a significant direct relation between myofascial release technique and modifications in the autonomic nervous system activity [22].

Regarding the interaction between OMT and tissue modification, in-vitro models highlight a possible decrease in the production of inflammatory factors [23].

A possible role of neuroendocrine factors can be hypothesized as indicated by the evidence of the effect of OMT on pain biomarker modification in patients affected by low back pain [24].

This study also shows that a significantly higher rate of premature infants receiving osteopathic care can be discharged before 28 days regardless of gender, GA, birth weight and oral feeding at admission.

Such a result may have important implications for the optimization of health care in premature infants. Focusing on the percentage of patients discharged before a given threshold, rather than looking at the average reduction in LOS, may be very relevant for health optimization and cost control. Reducing the rate of long stays would reduce the number of patients in the NICU, allowing for more cribs to become simultaneously available for those infants who require specialized care.

From an epidemiological point of view, the potential benefit may also spread beyond discharge, considering that hospitalization can influence nutrition [25] and morbidity of gastrointestinal infections [26].

An understanding of the differential advantage of OMT on specific subgroups, in particular within specified classes of GA, will require ad hoc studies with an adequate sample size. In the present study, it was not possible to perform subgroups analyses on subjects with very low GA, due to the very limited number of patients available for enrollment.

Finally, some intrinsic limitations of the present study need to be outlined.

This report is based on measurements implemented at the local NICU at the start of the study. Additional relevant confounding variables such as maternal/delivery factors (including breast feeding), respiratory support, method of feeding and gastric emptying time could not be included in this study.

Treatment allocation was neither randomized nor structured, as it was based on matters of convenience within the constraints of the proposed two days per week of osteopathic care. Furthermore, due to the current logistics and procedures it was not possible to "blind" nurses and neonatologists to treatment regimen.

This study, which was conducted in only one NICU, cannot capture the intrinsic variability of organizational strategies across multiple clinical centers managing the complexities of the overall population of newborn infants.

From a methodological point of view, sample size was not based on formal power estimation, treatment was not allocated using a random procedure, and the population of preterm infants may not be representative of the entire population of cases.

The above limitations affect our ability to check for bias and duly rely on the precision of our estimates. In other terms, both the size of the effect of OMT (point estimate) and its level of uncertainty (95% confidence interval) are more likely to be inconsistent with further results obtainable under more general conditions.

To evaluate the efficacy of OMT more studies are required using formal experimental methods, such as randomized and placebo controlled clinical trials. The best endpoint of a well designed three armed study would be the difference between the sham and the actual treatment. However, to make it possible, osteopaths should collaborate with NICU managers to revise the application of operational procedures, so that OMT can be smoothly applied on large populations, across multiple clinical sites.

Despite the above limitations, and given the current lack of information on the possible effects of OMT in preterm infants, the finding of this report sets an interesting ground for new developments. Among these, the standard measurement of all relevant parameters represents an essential aspect that deserves attention for future investigations. Key characteristics and outcomes that can be easily monitored on a daily basis by clinicians, nurses and even parents of preterm infants have been identified. Their adoption for the construction of electronic data base registers can offer a sustainable means to improve both analysis and management of NICU activity, allowing to carry out more detailed exploratory studies while providing a basis for ongoing trials.

## Conclusion

The study suggests that osteopathic treatment may reduce a high occurrence of gastrointestinal symptoms and the rates of long-term stays.

Randomized control studies are needed to confirm these results and to generalize them to a broader population of high risk newborns.

## Abbreviations

NICU: neonatal intensive care unit; VLBW: very low birth weight; LBW: low birth weigh; GA: gestational age; LOS: length of stay; GER: gastroesophageal reflux; GR: gastric residual; OMT: osteopathic manipulative treatment; OR: odd ratio.

## Authors' contributions

GB and CD conceived the idea of the study. GP, GB, VC, CR and FCE participated in the design of the study and its coordination. GP, PT, MD, FCE and PF coordinated and performed the data collection. GP and FCA drafted the manuscript. FCA performed the statistical analysis. All authors read and approved the final manuscript.

## Competing interests

The authors declare that they have no competing interests.
